# Effect of Ammonia-Soda Residue on the Strength and Chloride-Resistance Performance of Steel Slag-Granulated Blast Furnace Slag-Based Concrete after Immersion in Artificial Seawater

**DOI:** 10.3390/ma14206048

**Published:** 2021-10-13

**Authors:** Chengwen Xu, Wen Ni, Keqing Li

**Affiliations:** 1 School of Civil and Resource Engineering, University of Science and Technology Beijing, Beijing 100083, China; isaac1964620@126.com; 2 Key Laboratory of the Ministry of Education of China for High-Efficient Mining and Safety of Metal Mines, University of Science and Technology Beijing, Beijing 100083, China; 3 Beijing Key Laboratory of Resource-Oriented Treatment of Industrial Pollutants, University of Science and Technology Beijing, Beijing 100083, China

**Keywords:** ammonia-soda residue, chloride resistance, BOFS–GBFS based concrete, resource reutilization

## Abstract

Ammonia-soda residue (ASR) is the main solid waste generated from soda manufacturing and is hard to reuse due to its complex chemical composition. This study investigated the influence of ASR content on the strength and chloride-resistance capacity of concrete based on basic oxygen furnace slag and ground blast furnace slag. The hydration and chloride resistance mechanisms were analysed by comparing the hydrate products and pore structural changes. The results showed that adding ASR had the greatest impact on early strength. ASR-introduced chloride ions may participate in the hydration process to generate Friedel’s salt and decrease ettringite. The optimum pore distribution appeared when the ASR-to-desulphurisation gypsum ratio was 2:3 because of the introduction of nucleation sites and the decrease of C–S–H gels. The two chloride resistance-capacity measurements were affected differently by the ASR content. The apparent chloride diffusion coefficient was mainly affected by the percentage of pores that were larger than 10 nm. However, electric flux increased when ASR increased due to the influence of introduced chloride. The crystallisation pressure of Friedel’s salt decreases the strength of concrete with ASR content after high-concentration artificial-seawater immersion. The significant chloride-resistance property provided an alternative use for the concrete containing ASR.

## 1. Introduction

**Figure 1:**
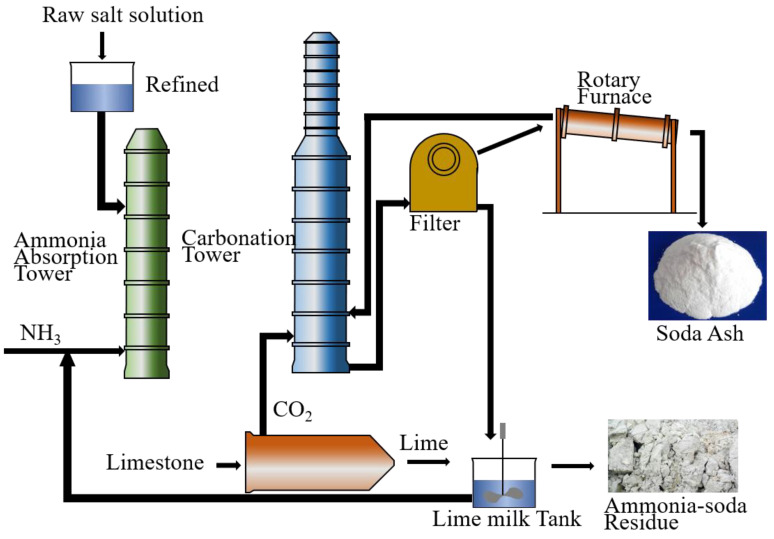
Generation process of ammonia soda ash and ammonia-soda residue.

The ammonia soda process, which is one of the most common soda-manufacturing processes, produced 14 million tons of soda ash in China in 2019 [1]. The emission of ammonia-soda residue (ASR) corresponding to this process was about 4.2 million tons [2,3,4]. Because of the wide utilization of soda ash, the generation of ASR increased with an increase in the soda ash production. The production process of ammonia soda ash and the generation of ASR are shown in Figure 1. ASR has a mud-like look due to the fine particle size and a large amount of water. The predominant mineral phases in ASR are NaCl, CaCl_2_, CaSO_4_, portlandite, calcite, and quartz [5]. An inappropriate manufacturing procedure would pollute the soil and groundwater due to the high alkalinity and ion concentration as Cl^−^ and SO_4_^2−^.

Although ASR might be used to produce engineering soil [6] and porous ceramic [7], the pretreatment of ASR and chloride volatilization under high temperature limited these applications. Considering the complex chemical composition and the solidification of Cl^−^, ASR was mostly used to produce cementitious materials and concrete. The addition of ASR could prevent concrete shrinkage due to the generation of ettringite [8]. The Cl^−^ would be captured by cement [9], GBFS [5,10], or fly ash [11] due to the generated Friedel’s salt or multiple gels. Although excess chloride content may raise the potential for reinforcement corrosion, the massive utilization of concrete could still improve the reuse of ASR.

Basic oxygen furnace slag (BOFS) and ground blast furnace slag (GBFS) were widely used to produce cementitious materials, BOFS–GBFS-based binder and BOFS–GBFS-based concrete [12,13,14,15]. With the proper addition, researchers demonstrated that ASR may accelerate the hydration rate and minimise the setting time of the BOFS–GBFS-based binder and the mechanical properties [16].

However, the chloride-resistance ability of concrete, which affected its use in corrosive environments, was not well-discussed in previous studies. The addition of GBFS could improve the chloride binding [17], perfect the pore structure [10], and inhibit charge transport in the binders [18] to reduce the chloride diffusion. But ASR may provide Cl^−^ at the beginning of hydration. Although BOFS–GBFS-based binders can bind some chloride in Friedel’s salt and C–S–H gels, the captured Cl^−^ may reduce the further chloride-binding capacity of paste in a chloride environment. On the other hand, the calcite introduced by ASR would influence the porosity of the binders [19]. Therefore, chloride-resistance capacity was essential to BOFS–GBFS-based concrete containing ASR.

Considering the CaSO_4_ and CaCl_2_ in ASR, ASR’s addition may also act as activators [5,20]. Therefore, the ASR-to-DG (desulphurisation gypsum) ratio was considered the main factor to investigate its influence on the concrete’s compressive strength and chloride-resistance capacity. The strength of the concrete was tested after being immersed in artificial seawater to predict its service performance. The phase changes of the paste were shown using X-ray diffraction (XRD) analysis (crystalline phase) and Fourier transform infrared spectroscopy (FTIR) analysis (bond variations). The pore structure and micromorphology of the paste were indicated through mercury intrusion porosimetry (MIP) and field-emission scanning electron microscopy (FE–SEM). The influence of ASR on hydration mechanism and chloride-resistance capacity was addressed. The use of BOFS–GBFS-based concrete containing ASR will increase with the illustration of chloride-resistance capacity in this study, reducing cement and the stockpiling of solid waste and protecting the environment.

## 2. Materials and Methods

### 2.1. Materials

**Figure 2:**
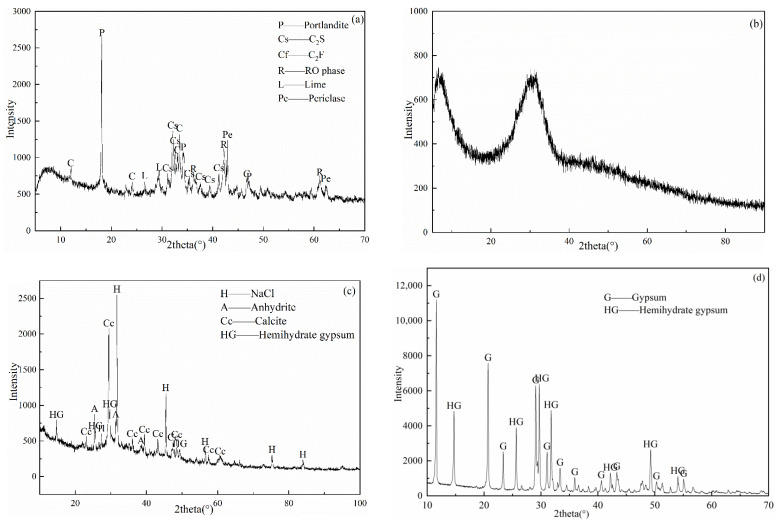
XRD patterns of the raw materials—(**a**) BOFS; (**b**) GBFS [13]; (**c**) ASR; and (**d**) DG.

The cementitious materials in this work consisted of GBFS (provided by Wuhan Iron and Steel Group Co., Wuhan, China), BOFS (also from Wuhan Iron and Steel Group Co.), DG (provided by Jintaicheng Environmental Resources Company Limited, Xingtai, China), and ASR (offered by Tangshan Sanyou Chemical industry Co., Ltd., Tangshan, China). The chemical composition and specific surface area are shown in Table 1. Figure 2 presented the mineral composition of the raw materials. The mineral phases of BOFS included portlandite, dicalcium silicate (C_2_S), dicalcium ferrite (C_2_F), lime, periclase, and RO phase, while the GBFS mainly presented as amorphous phases. ASR mainly consisted of calcite, halite, gypsum, and hemihydrate gypsum. All of the raw materials were oven-dried before further treatments. The GBFS, BOFS, and DG were ground into powders with the specific surface areas in Table 1, and the ASR was crushed into less than 1 mm.

The iron tailings were provided by Beijing Miyun Weike Mining Co., Ltd. (Beijing, China) as coarse (5–25 mm) and fine (0.08–5 mm) aggregates. A polycarboxylate superplasticiser was also applied to adjust the concrete mix fluidity.

### 2.2. Sample Preparation

The mix proportion of the concrete is shown in Table 2. Considering the similarity of DG and ASR in reaction with the BOFS–GBFS-based binder [16], the ASR-to-DG ratio was the main factor, with four levels, 1:3, 2:3, 3:3, and 3:2, in the mix proportion. The density of the concrete was 2400 kg/m^3^. Based on previous studies [12], the BOFS-to-GBFS ratio was determined to be 1:4. The sand ratio was 0.5, and the water/binder ratio was 0.28.

The concrete samples were produced on the basis of the proportion in Table 2. The concrete was demolded after 24 h. The curing condition, 20 °C and 90% relative humidity, satisfied the Chinese standard GB/T 50081–2002 for a test method for mechanical properties on ordinary concrete [21]. The sample dimensions of 100 mm × 100 mm × 100 mm were cured at 3, 28, 60 and 180 days and tested to measure the compressive strength. The cylinder concretes with dimensions of Φ100 × 50 mm were prepared to test electric flux and chloride penetration. A paste with the same proportion as concrete was produced and cured under the same conditions to conduct mechanism analysis. After 180 days’ curing, the paste was immersed in anhydrous alcohol to terminate hydration. Before being ground into powder, the paste was crushed and dried by a vacuum oven. There was also a pasted piece to observe the microstructure.

### 2.3. Methods

The compressive strength (average of three samples) was measured using cubic concrete after 3, 28, 60 and 180 days curing according to the Chinese standard GB/T 50081–2002. A high concentration of artificial seawater was prepared (Table 3) to study the concrete properties in marine water. After 28 days of curing, the cubic samples were immersed in artificial seawater. Once a month, the artificial seawater was replaced. The immersed cubic concrete was tested after being put into the solution for 152 days to study the strength change after being immersed in the artificial seawater.

Conductivity and chloride penetration in a high-concentration solution was used to measure chloride resistance. The conductivity was determined using an electric flux according to the Chinese standard GB/T 50082-2009 for the long-term performance and durability of ordinary concrete [22]. The concrete cylinders were placed in a vacuum water saturation instrument for 22 h to achieve saturation. The water on the concrete’s surface was wiped away. The conductivity was measured using an HC-RCMP6 combined measuring instrument for the chloride diffusion coefficient and the electric flux of concrete purchased from CABRhuaCe (HangZhou) Science and Technology Co., Ltd. (Hangzhou, China). During the measurement, a NaCl solution with a mass fraction of 3.0% and NaOH solution with a concentration of 0.3 mol/L was used. The average of three samples’ conductivity was taken as the result.

To investigate the penetration process of chloride ions, two cylindrical samples of each group with epoxy resin-coated sides and bottoms were immersed in high-concentration artificial seawater (Table 3) for 28 days. The concrete was cut into pieces from the exposed surface to a 25 mm depth after 152 days of immersion. The layers were 3, 3, 4, 5, 5, and 5 mm thick. The chloride concentration of each layer was tested based on the ASTM standard Determining the Apparent Chloride Diffusion Coefficient of Cementitious Mixtures by Bulk Diffusion [23].

The phase change of the paste powder was measured using an X-ray diffraction analysis with a scan angle of 5° to 90° and a step of 0.02°. Rigaku Co. (Tokyo, Japan) provided the XRD instrument with a copper target and an Ultima IV diffractometer. The bond transformation of the paste was evaluated using Fourier transform infrared spectroscopy. Shimadzu Co. (Kyoto, Japan) provided the spectrometer with a model FTIR-8400s. The wavenumber varied from 400 to 4000 cm^−1^. The paste piece with a diameter > 5 mm was used to conduct the mercury intrusion porosimetry with an Autopore IV 9510 mercury porosimeter. The instrument was provided by Micromeritics Instrument Co. (Norcross, GA, USA). The microstructure and morphology of the hydrate products were studied using field-emission scanning electron microscopy. The lamellar paste was coated with gold to enhance conductivity. Zeiss Co (Oberkohen, Baden-Württemberg, German) provided the SUPRA-55 field-emission scanning electron microscope used in this analysis. Dispersive-energy X-ray (EDX) spectroscopy was used to determine the element composition.

## 3. Results

### 3.1. Compressive Strength

**Figure 3:**
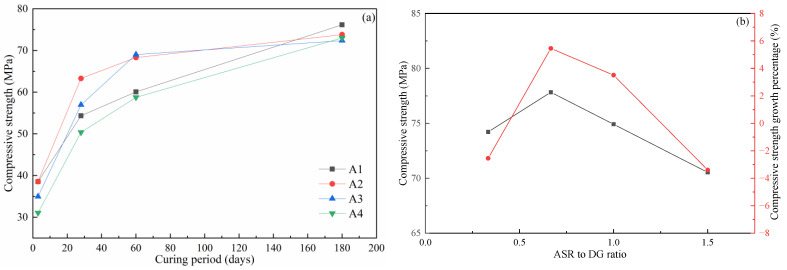
Compressive strength and strength growth of concrete: (**a**) the compressive strength of concrete in different curing period and (**b**) compressive strength and strength growth percentage of concrete after artificial seawater immersing.

The compressive strength of the concrete is shown in Figure 3a. Within 60 days, the ratio of ASR to DG had an obvious effect on the compressive strength of concrete. At the early stage of hydration (within 3 days), the compressive strength of concrete decreased with the increase of the ASR-to-DG ratio once the ASR-to-DG ratio exceeded 2:3. Within 60 days of increasing the curing time, the strength growth of 2:3 and 3:3 concrete groups was the most noticeable. It can be seen that, in the early stage of hydration (within 3 days), DG played a more significant role in strength growth than ASR. However, the maximum at 2:3 limited strength growth. In the middle stage of curing, the compressive strength of concrete was affected by the dual factors of ASR and DG. The optimal ratio of ASR to DG was between 2:3 and 3:3. When cured for 180 days, the strength of each proportion of concrete stayed at a close level and achieved 76.2, 73.8, 72.4, and 73.0 MPa, respectively.

Figure 3b shows the compressive strength after 152 days of immersion and its growth percentage compared to concrete in standard conditions The compressive strength of concrete for four ASR to DG ratios is 74.2, 77.8, 74.9, and 70.5 MPa, respectively, with the ASR-to-DG ratio first increasing and then decreasing. The strength growth percentage followed a similar pattern as compressive strength. The growth percentage also reached its maximum at an ASR-to-DG ratio of 2:3. Compressive strength was lower than 2.6% when cured in standard conditions with a low ASR-to-DG ratio (1:3). The significant growth percentage increased to 5.5% and 3.8% after the ASR-to-DG ratio reached 2:3 and 3:3. A higher ASR-to-DG ratio also caused the strength to decrease after being immersed in artificial seawater.

### 3.2. Electric Flux Analysis

**Figure 4:**
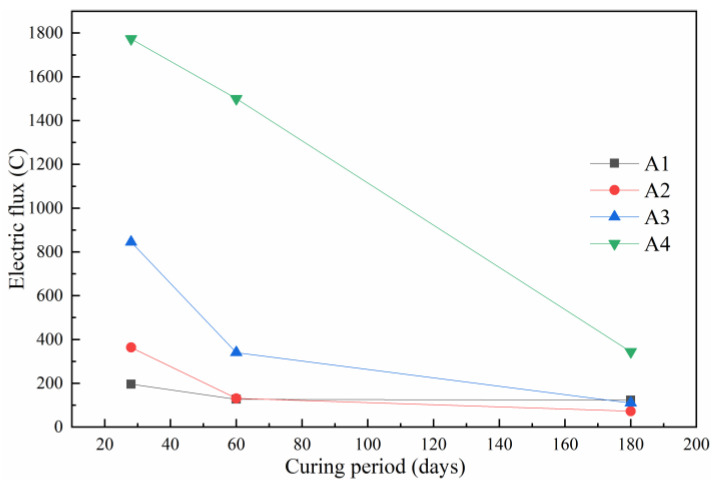
Electric flux of concrete at different ages.

The electric flux was chosen to represent the conductivity change based on the conductivity measurement. A better conductivity indicated that the concrete transported more chloride ions under the same voltage, which indicated poor chloride resistance. The results are shown in Figure 4. The increase in the ASR-to-DG ratio had a significant positive effect on the growth in concrete electric flux. The electric flux values of the concrete at 28 days were 1774 C, 845 C, 364 C, and 196 C, respectively, as the ASR-to-DG ratio decreased. When the ratio of ASR to DG was lower than 3:3, the electric flux of concrete decreased significantly from 28 to 60 days. The electric flux of these three groups decreased to 340 C, 132 C, and 127 C, respectively. The electric flux of concrete with an ASR-to-DG ratio of 3:2 decreased slightly. With the extension of curing time, the electric flux of concrete with an ASR-to-DG ratio of 3:2 decreased significantly to 344 C from the 60 days to 180 days, while the other three groups remained below 125 C, although the reduction was small. The increase in ASR content had a more obvious impact on concrete before 60 days in curing. The problem of increasing concrete electric flux caused by an increase in ASR content could be mitigated to some extent with the increase in hydration products and the optimisation of concrete structure as the curing time is extended. However, the effect of excessive ASR content on concrete was limited.

### 3.3. Chloride Profile Analysis

**Figure 5:**
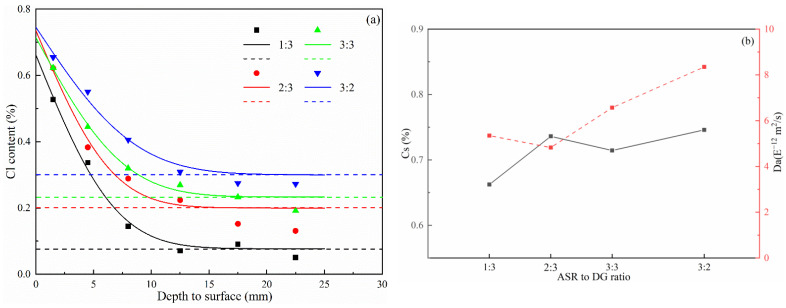
Chloride penetration results of concrete after 152 days of immersion—(**a**) chloride profiles of the concrete and the (**b**) Cs and Da of the concrete.

Figure 5a shows the variation in the chloride ion content with concrete depth after being immersed in high-concentration artificial seawater under various ASR-to-DG ratios. The dashed line shows the initial chloride ion content of the concrete samples (tested based on [23]). Figure 5a shows that, with an increase in the ASR-to-DG ratio, the initial chloride ion concentration of concrete gradually increases, with values of 0.07%, 0.20%, 0.23%, and 0.30% respectively. The penetration depth of chloride ion slightly increased with the increase of the ASR-to-DG ratio and was stable between 12.5–17.5 mm. The chloride ion concentration in each layer of concrete also increased significantly when the ASR-to-DG ratio increased, due to the increase in initial chloride ion concentration. When the ratio of ASR to DG was higher than 2:3, the chloride ion concentration in the concrete surface (1.5 mm depth) did not significantly increase and was maintained at 0.62–0.64%. However, the chloride ion concentration in the interior increased significantly.

Based on Fick’s second law, the chloride ion concentration data in Figure 5a are fitted based on Equation (1).

(1) $$C\left(x,\;t\right)=\;\mathrm{Cs}\;-\left(\mathrm{Cs}\;-\;\mathrm{Ci}\right)\times \;\mathrm{erf}(x/\sqrt{4\;\times \;\mathrm{Da}\;\times \;t})$$

The C(x, t) represents the chloride concentration at depth x and time t. Cs is the surface chloride ion concentration, and Ci is the initial chloride-ion concentration of the samples. Da is the apparent chloride diffusion coefficient, and erf is the error function. The fitting curve is shown in Figure 5a, and the Cs and Da are shown in Figure 5b. It can be observed that, with an increase in the ASR-to-DG ratio, the Cs of concrete is 0.66%, 0.74%, 0.71%, and 0.75% respectively, showing an overall upward trend. After eliminating the impact of the initial chloride ion concentration, it could be concluded that the Cs for the four ASR-to-DG ratios were 0.59%, 0.54%, 0.48%, and 0.45%, respectively, which decreased with the increase of the ASR-to-DG ratio. Overall, when the ASR-to-DG ratio increased, the Da of concrete decreased slightly and then gradually increased, reaching a minimum value of 4.83 × 10^−12^ m^2^/s when the ASR-to-DG ratio was 2:3 and the maximum value of 8.35 × 10^−12^ m^2^/s when the alumina-soda residue-to-gypsum ratio is 3:2.

### 3.4. XRD Analysis

**Figure 6:**
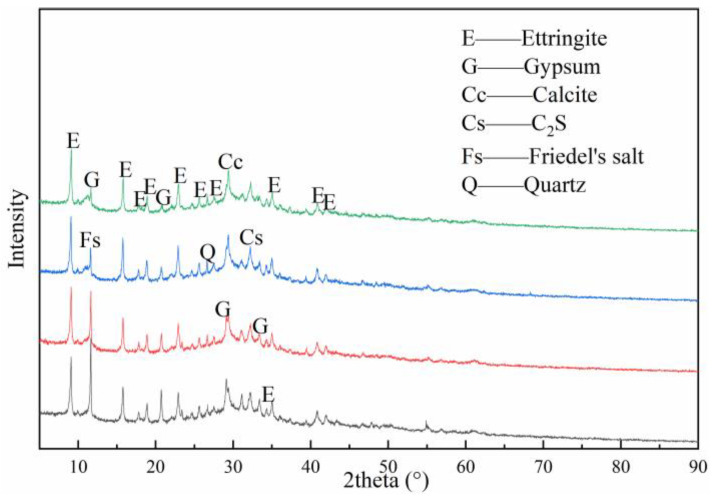
XRD patterns of the paste with different ASR-to-DG ratios at 180 days.

Figure 6 shows the XRD patterns of paste powder with various ASR-to-DG ratios after 180 days. The main mineral components of the pastes are ettringite, gypsum, calcite, C_2_S, Friedel’s salt, and quartz. Despite the gypsum, calcite, and C_2_S coming from raw materials (DG, ASR, and BOFS, respectively [12,16]), ettringite and Friedel’s salt were the products generated during hydration. The diffraction peak of gypsum in pastes gradually decreased as the ASR-to-DG ratio increased after 180 days, whilst the peak of calcite steadily increased. This was mainly attributed to the increase of ASR and the decrease of DG. The peak of ettringite first increased and then decreased with an increase in the ASR-to-DG ratio, reaching a maximum when the ASR-to-DG ratio was 3:3. However, the change range was small. The peak of Friedel’s salt was enhanced with the increase in the ASR-to-DG ratio, indicating that the increase in ASR promotes the formation of Friedel’s salt. There is a certain competitive relationship between Cl^−^ and SO_4_^2−^ in the hydration process. Both of them can react with alumina octahedron produced by GBFS decomposition to produce Friedel’s salt and ettringite, respectively [16,24]. With the content of ASR increasing, the concentration of Cl^−^ in the pastes increases, which is favourable to the formation of Friedel’s salt. The competition between Cl^−^ and SO_4_^2−^ and the decrease of SO_4_^2−^ concentration caused by the decrease in DG content slightly reduced the formation of ettringite together.

### 3.5. FTIR Analysis

**Figure 7:**
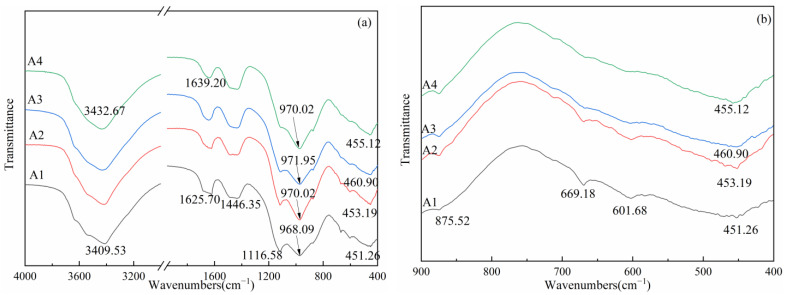
FTIR spectra of the samples with different ASR-to-DG ratios at 180 days: (**a**) full-range FTIR; (**b**) range from 400 to 900 cm^−1^.

Figure 7 shows the FTIR spectra of powders with different ASR-to-DG ratios. The generation of the hydrate products was revealed by the peaks at 3409.53–3432.67 cm^−1^ and 1625.70–1639.20 cm^−1^, which belong to the O–H bond deformation and stretching vibrations [25,26], respectively, according to the full range spectra in Figure 7a. With the increase in the ASR-to-DG ratio, those bonds became slightly narrow, indicating that the hydrate products decreased with the proportion change. The band at approximately 970 cm^−1^ was attributed to the internal stretching vibrations of the Si–O–Si bond in C–S–H gels [27]. With the increase in the ASR-to-DG ratio, the peak moved to a higher wavenumber at first, indicating an increase in the polymerization degree, and then decreased. The highest wavenumber (971.95 cm^−1^) appeared at the A3 group. Figure 7b shows detailed information on wavenumbers between 400 and 900 cm^−1^. When the two spectra were combined, there were two bands at 1116.58 and 601.68 cm^−1^, which were correlated with the asymmetry and symmetry vibrations of SO_4_^2−^ from gypsum and ettringite respectively [28]. These two peaks became weaker as the ASR-to-DG ratio increased. The band at 875.52 was related to the Al–O–H bond referring to the alumina octahedron [28]. It was sharper with the addition of ASR content. The peak at 669.18 cm^−1^ was attributed to the Si–O–Al bond in GBFS [29]. The disappearance of this peak during the increase in the ASR-to-DG ratio indicated the increased bond rupture. Furthermore, the calcite in ASR was responsible for the peak at 1446.35 cm^−1^ [30], which became slightly sharper with the ratio increasing.

### 3.6. Pore Structure

**Figure 8:**
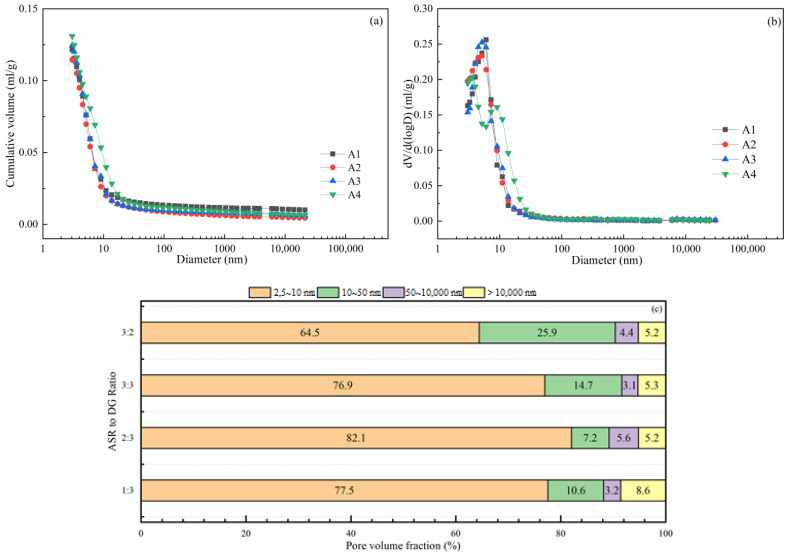
Pore volume and distribution of samples with different ASR-to-DG ratios at 180 days—(**a**) cumulative pore volume, (**b**) dV/d(logD), and (**c**) pore-volume fraction distribution.

Figure 8 shows the pore volume and distribution of binders at 180 days with various ASR-to-DG ratios. The porosity and pore-size distribution of the hardened paste have a significant impact on prepared concrete strength and Cl^−^ permeability. The formation of hydration products gradually occupied the water space in the hydration-hardened slurry, dividing the space into irregular pores. The pores in the hardened slurry can be divided into micropores (<2 nm), mesopores (2–50 nm), and macropores (>50 nm) [31]. Mesopores can be subdivided into two pore size ranges: 2–10 nm pores, which do not affect the strength and permeability but affect shrinkage, and 10–50 nm pores that affect the strength, permeability, and shrinkage [32,33]. The total pore volume of cementitious-material-hardened slurry first decreased and then increased when the ASR-to-DG ratio increased. The minimum value of 0.115 mL/g was attained when the ASR-to-DG ratio was 2:3. When the ratio of ASR to DG reached 3:2, the cumulative pore volume in the pore-size range of 5–17 nm was much higher than that in the other three groups. The fractal dimension of the pore volume in Figure 8b shows that, when the ratio of ASR to DG was lower than 3:3, there was only one most probable pore diameter in the pore distribution of binder, and that was between 5–6 nm. When the ratio of ASR to DG increased to 3:2, there were two pore diameters in the hardened slurry, which were 3.62 nm and 9.05 nm, respectively. When the pore diameter was greater than 9.05 nm, the pore volume of each pore diameter was larger than the other three ratios.

Figure 8c shows the pore-volume fraction distribution. The pore proportion in different pore-size ranges also showed the same variation law with dV/d(logD): when the ratio of ASR to DG is 2:3, the paste contained the largest volume fraction of harmless pores (<10 mm) and the smallest volume fraction of large pores (>10 mm). Above or below this ratio, the volume proportion of harmless holes gradually decreased, and the proportion of harmful holes presented an opposite trend. Among the harmful pores in the three pore sizes, the harmful pores of 10–50 nm were more affected by the change of the ASR-to-DG ratio, with proportions of 10.6%, 7.2%, 14.7%, and 25.9%, respectively. Harmful pores with pore diameter above 50 nm were less affected, and the proportion of different ASR-to-DG ratios was about 10%.

Based on the porosity of hardened paste under the different ASR-to-DG ratios in Table 4, with the increase in the ASR-to-DG ratio, the porosity of cementitious material decreased first and then increased. The porosity reached the minimum when the ASR-to-DG ratio was 2:3, similar to the change law of total pore volume.

### 3.7. FE-SEM Analysis

**Figure 9:**
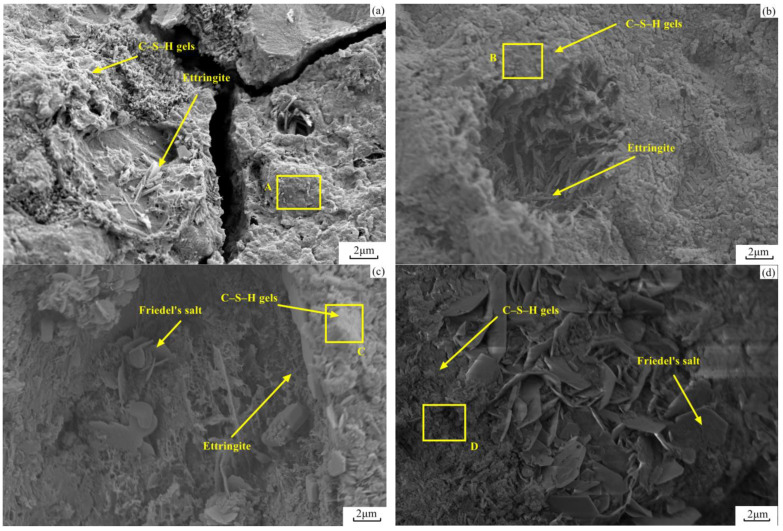
Micromorphology of a paste of different ASR-to-DG ratios at 180 days: (**a**) A1, (**b**) A2, (**c**) A3, and (**d**) A4.

The micromorphology of paste pieces with different ASR-to-DG ratios at 180 days is shown in Figure 9. Ettringite, C–S–H gels, and Friedel’s salt were observed in the pores or cracks of the binders. Other hydrate products like ettringite and Friedel’s salt were covered by C–S–H gels, which was considered the reason for the strength improvement. The hydrate product mainly included ettringite and C–S–H gels at low ASR content. When the ASR-to-DG ratio increased, a crystal of Friedel’s salt appeared in the pores of the A3 and A4 pastes, caused by the gypsum participating in the hydration reaction. The further ratio increase was also responsible for Friedel’s salt’s crystal growth compared to the crystal size in Figure 9c,d.

**Figure 10:**
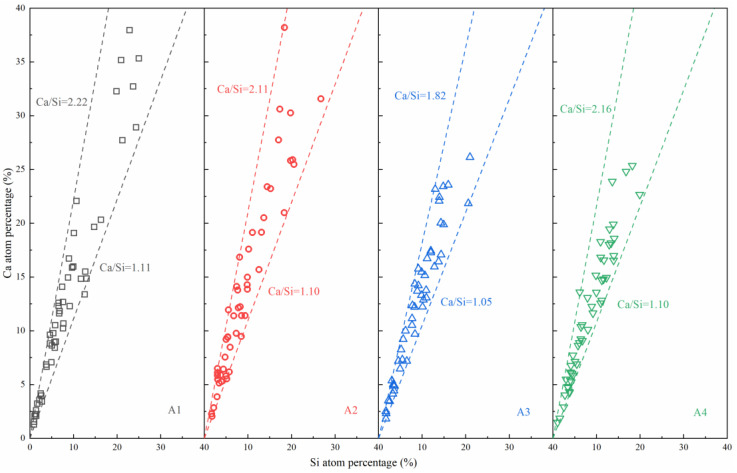
Atom percentages of Ca and Si in Figure 9.

To investigate the difference in C–S–H gels between four pastes, fifty points were selected in areas A, B, C, and D of Figure 9. The atom percentages of Ca and Si were determined using EDX analysis, to plot the scatter diagrams in Figure 10. The Ca/Si percentages could reveal the C–S–H gel structure and mechanical properties. With a lower ratio of Ca to Si, the C–S–H gels presented a longer chain structure and better mechanical properties [34,35]. Every Ca/Si of the C–S–H gels in four pastes maintained a certain range. With different ASR-to-DG ratios, the Ca/Si ranges were 1.11–2.22, 1.10–2.11, 1.05–1.82, and 1.10–2.16, respectively. The content increase of ASR reduced the atom ratio of Ca/Si until group A3. Therefore, the mechanical properties of C–S–H gels in A3 show slightly better properties than in the other three binders.

## 4. Discussion

### 4.1. Influence of ASR Content on the Hydration Mechanism

**Figure 11:**
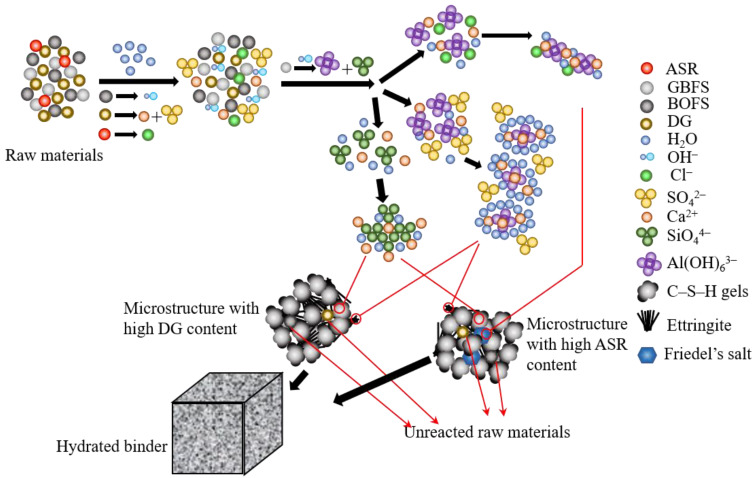
Hydration mechanism of binders with ASR content.

The hydration mechanism of BOFS–GBFS-based binders with DG addition has been described previously [12,36]. The hydration mechanism of binders with ASR replacing DG is shown in Figure 11. Under low ASR content, it barely had influence on the hydration process. The presence of BOFS provided the OH^−^ needed to accelerate the dissolution of GBFS, which was confirmed by the Si–O–Al bond being broken in Figure 7. With the Ca^2+^ and SO_4_^2−^ provided by BOFS and DG, C–S–H gels and ettringite were generated to provide the strength source [37,38]. The further addition of ASR introduced more Cl^−^ in the system; with the alumina octahedron provided by GBFS, Friedel’s salt was generated as Equation (2) [16]:

4Ca^2+^ + 2Cl^−^ + 2Al(OH)_6_^3−^ → Ca_4_Al_2_(OH)_12_Cl_2_·4H_2_O (2)

Similar with SO_4_^2−^, Cl^−^ also reacted with alumina octahedron. Therefore, there is a competition between SO_4_^2−^ and Cl^−^ that was impacted by the increase of ASR content. The generation of ettringite and Friedel’s salt could reveal this competition. Although the low solubility of ettringite provided a higher reaction possibility of SO_4_^2−^, this competition was still influenced by ASR content. With an increase in the ASR content, the total formation of Friedel’s salt and ettringite increased according to the Al–O–H bond becoming sharper in FTIR spectra. However, Friedel’s salt increased with the slight decrease of ettringite based on the XRD and FE–SEM analyses.

The formation of C–S–H gels was also affected by the addition of ASR content. The content of ASR introduced calcite and Cl^−^ in the system. At the beginning of the hydration, calcite could provide nucleation sites to form C–S–H gels [19]. The GBFS dissolution accelerated by Cl^−^ reacting with alumina octahedra could provide silica tetrahedra to participate in the generation of C–S–H gels. However, the increase of ASR slightly decreased the generation of C–S–H gels according to the FTIR spectra in Figure 7. It can be indicated that DG plays a more important role in the bond breaking of GBFS during the middle and late stages of hydration. Therefore, there were more silica tetrahedra formed C–S–H gels after 180 days in a paste with high DG content. Besides, the increase of ASR content decreased the Ca/Si ratio of C–S–H gels first and then increased. The strength of binders was also influenced due to the higher mechanical properties provided by C–S–H gels with a low Ca/Si ratio.

### 4.2. Effect of ASR on Chloride Penetration of Concrete

The porosity of concrete was one of the most fundamental factors that influenced the chloride penetration process [39,40,41]. The added calcite could improve the porosity to promote porosity with the increase of ASR content [19]. However, the generation of C–S–H gels was also reduced, negatively impacting the pore structure. Combining these two factors, the MIP analysis revealed that the increase of ASR content improved the porosity at first. A further increase would raise the porosity and improve the pore percentage, which provides the intrusion path for Cl^−^.

Furthermore, the chloride-binding capacity also affected the chloride penetration [39]. The binding capacity included two aspects: the alumina octahedron reacting with Cl^−^ to form Friedel’s salt and C–S–H gels capturing Cl^−^. The FTIR results in Figure 7 proved that the increase in ASR improved the generation of alumina octahedron. It can be stated that the binders that contained low ASR content could provide more alumina octahedron during the Cl^−^ penetration. Besides, the low ASR binder also formed a slightly higher C–S–H gel content to capture Cl^−^. Therefore, the chloride-binding capacity decreased with the increase of ASR content, as illustrated by the chloride profiles.

The influence of ASR on the two chloride-resistance capacity methods was slightly different. Based on chloride profiles, the apparent chloride diffusion coefficient of the concrete met the changing pattern of pores larger than 10 nm. It can be stated that the penetration of high-concentration chloride solution was mainly affected by the pore structures. However, under an external source, chloride introduced by ASR may participate in the transportation of Cl^−^ in solution according to the electric flux increase.

### 4.3. Effect of ASR on the Strength Change of Concrete after Artificial Seawater Immersion

The porosity of binders reduced first, then increased as the ratio of ASR to DG increased. Also, the proportion of 10–50 nm pore size, which affected strength and permeability [31], also decreased first and then increased. Thus, the concrete strength change law was also affected. Simultaneously, the concrete immersed in high-concentration artificial seawater was affected by chloride ion intrusion, which presented a similar trend with the pore distribution. With a high concentration of Cl^−^ intrusion, Friedel’s salt’s formation might have occurred in the pores and caused crystallisation pressure, which was responsible for the strength decrease [42]. Therefore, the concrete with low porosity and better pore distribution presented a better strength performance after immersing in high-concentration artificial seawater.

## 5. Conclusions

This paper focused on the influence of ASR on the strength and chloride-resistance capacity of BOFS–GBFS-based concrete. The mechanism of strength change and electric flux was also discussed using XRD, FTIR, MIP, and FE–SRM analyses.

The addition of ASR strongly affected the early strength of the concrete. After 180 days’ curing, the strength of the concrete showed a similar lever of 73 MPa. With the immersion into high-concentration artificial seawater, the strength of the concrete decreased at a high and low ASR content, while concrete with the ASR-to-DG ratio of 2:3 and 3:3 presented strength improvement. The electric flux of the concrete increased with the rising ASR content. Due to the pore structure caused by extending the curing time, the electric flux decreased gradually.

Like the BOFS-GBFS-based binders without ASR, the mainly hydrate product included ettringite and C–S–H gels. The increase in ASR content improved Friedel’s salt generation and slightly decreased the amount of ettringite. The total amount of them increased at the same time. Meanwhile, the formation of C–S–H gels also decreased slightly. But the mechanical properties of C–S–H gels achieved a maximum at a 3:3 ASR-to-DG ratio due to the highest Ca/Si ratio.

The ASR content had both positive (due to the calcite introduced) and negative (due to the formation reduction of C–S–H gels) influence on the paste porosity and pore structure. The improvement of ASR content decreased the chloride-binding capacity of the concrete. The influence of ASR content on the two chloride-resistance capacity measurements was different. Da was mainly affected by the pore percentage that was larger than 10 nm. However, the electric flux decreased with increasing ASR content. Besides, due to the higher bound Cl^−^, the crystallisation pressure of Friedel’s salt was responsible for the strength loss at low and high ASR content.

The introduction of ASR to a BOFS–GBFS-based cementitious system extended the range of solid as an activator. With considerable chloride-resistance capacity and strength performance in a high-chloride environment, the utilization of concrete containing ASR was improved. Therefore, economic and environmental benefits due to the utilisation of ASR, BOFS, GBFS, and DG include reducing cement demand, decreasing solid waste stockpiling, and preventing soil and water solution.

## Figures and Tables

**Table 1:** Chemical composition and specific surface area of raw materials.

	GBFS	BOFS	DG	ASR
Chemical composition (%)	-	-	-	-
SiO_2_	35.03	14.17	1.58	12.99
Al_2_O_3_	15.73	4.30	0.53	3.25
Fe_2_O_3_	1.07	26.32	0.38	1.17
CaO	36.61	39.96	39.15	44.92
MgO	8.59	8.22	0.32	7.96
SO_3_	0.09	0.18	36.22	5.67
K_2_O	0.01	0.01	-	0.38
Na_2_O	0.30	0.07	-	4.20
P_2_O_5_	-	1.29	-	-
Cl^−^	-	-	-	19.11
The specific surface area (m^2^/kg)	560	450	420	-

**Table 2:** Mix proportion of the concrete (kg/m^3^).

Group	Cementitious Materials				Aggregates		Water	Plasticizer
	GBFS	BOFS	DG	ASR	Fine	Course		
A1	320	80	75	25	880	880	140	1.92
A2	320	80	60	40	880	880	140	1.92
A3	320	80	50	50	880	880	140	1.92
A4	320	80	40	60	880	880	140	1.92

**Table 3:** Chemical proportion of a concentrated artificial seawater (g/L).

Compound	NaCl	MgCl_2_	CaCl_2_	Na_2_SO_4_	NaHCO_3_	KCl
Concentration	122.65	26.00	5.80	20.45	1.01	3.48

**Table 4:** Porosity variation of different ASR-to-DG ratios at 180 days.

Group	ASR-to-DG Ratio	Porosity (%)
A1	1:3	21.34
A2	2:3	21.06
A3	3:3	22.03
A4	3:2	22.75

## Data Availability

The data presented in this study are available on request from the corresponding author.
